# Identification and trajectory of multimorbidity patterns among older people in China: a longitudinal study based on the China health and retirement longitudinal study 2011–2020 data

**DOI:** 10.3389/fpubh.2025.1597224

**Published:** 2025-07-21

**Authors:** Qian Liu, Shuzhi Lin, Lin Yin, Wei Liu, Xiaoying Zhu, Zimeng Li, Yifang Shen, Bianling Feng

**Affiliations:** ^1^The Department of Pharmacy Administration, School of Pharmacy, Xi'an Jiaotong University, Xi’an, China; ^2^The Center for Drug Safety and Policy Research, Xi'an Jiaotong University, Xi’an, China

**Keywords:** multimorbidity, older populations, disease trajectory, public health, China

## Abstract

**Background:**

Multimorbidity presents a significant global health challenge, particularly among older people in China; however, research on its patterns and dynamic evolution remains limited. This study examines chronic disease co-occurrence and associated risk factors, identifying multimorbidity trends to inform health management and policy.

**Methods:**

This study utilized the latest five-wave national survey data from the China Health and Retirement Longitudinal Study (CHARLS) on 2,798 individuals aged 60 years or older with multimorbidity. Latent class analysis (LCA) identified distinct multimorbidity patterns from 14 self-reported chronic conditions. Longitudinal follow-up data were then used to construct transition frequency matrices, modeling the trajectory and dynamic evolution of multimorbidity among older people in China over time.

**Results:**

Using LCA, 2,798 participants were classified into four patterns: the multi-system disorder group (10.33%), the gastrointestinal metabolism group (44.07%), the cardiovascular disease group (37.81%), and the respiratory disease group (7.79%). Over five waves, the gastrointestinal metabolism group declined (from 74.70% in 2011 to 44.07% in 2020), while the cardiovascular group increased (from 15.87 to 37.81%). The multi-system and respiratory groups also grew. Transition analysis showed the gastrointestinal metabolism group was the least stable, with many shifting to the cardiovascular group (7.29% from 2013 to 2015, 23.81% from 2015 to 2018, 18.54% from 2018 to 2020).

**Conclusion:**

The disease burden among older people is shifting from gastrointestinal metabolism disorders to cardiovascular diseases, with gastrointestinal issues potentially acting as precursors to cardiovascular conditions. Future research should investigate the risk factors influencing transitions between multimorbidity patterns and their underlying mechanisms.

## 1 Introduction

Multimorbidity, typically defined as the coexistence of two or more chronic conditions in an individual, has emerged as a critical global public health issue, posing significant challenges to healthcare systems worldwide (1, 2). Research shows that the risk of multimorbidity increases substantially with age, contributing to higher mortality rates, functional decline, reduced medication adherence, and escalating healthcare costs among the aging population (3–5). Thus, studying multimorbidity is crucial for understanding the complexity of health among older people and alleviating the burden on healthcare systems (6).

China has the largest population of older people globally, 75.8% of whom suffer from at least one chronic condition (7). After age 60, multimorbidity prevalence rises sharply, affecting over 30% of older people, and reaching 90% in rural areas (5, 8). Widespread multimorbidity significantly impedes efforts to achieve healthy aging (2, 9). Despite the global focus on chronic diseases, most studies still concentrate on single-disease treatments, or treat multimorbidity merely as a sum of chronic conditions, leaving substantial gaps in the understanding of multimorbidity (10–12).

Chronic diseases rarely occur in isolation but tend to cluster due to shared risk factors, forming distinct multimorbidity patterns (13–15). Understanding these patterns, rather than treating each condition separately as in traditional clinical practice, may improve overall treatment efficacy (16). Common multimorbidity patterns among older people include cardiovascular, respiratory-metabolic, mental health, and non-specific disease clusters (17, 18). However, most studies rely on short-term or cross-sectional data and neglect long-term dynamics (1, 19). Moreover, differences in study populations, methodologies, and disease classifications limit the generalizability of findings, particularly in rapidly aging regions like China (20).

Few studies in China have utilized the most recent national longitudinal data to examine multimorbidity patterns and changes over time (16). Existing studies often rely on outdated datasets, failing to reflect the latest health trends. As a result, the long-term evolution of multimorbidity patterns in China remains unclear. For example, a study using 2015 data from the China Health and Retirement Longitudinal Study (CHARLS) identified 72 dual and 169 triple disease combinations, but did not explore underlying mechanisms or risk factors, limiting its practical relevance (10). Similarly, a study using 2018 data from the Chinese Longitudinal Healthy Longevity Survey (CLHLS) examined chronic disease clusters; however, this study did not capture recent changes in older peoples’ health, thus limiting its value for current policy and clinical interventions (21). Furthermore, unlike developed countries with more advanced multimorbidity management strategies, China has yet to establish guidelines for managing different multimorbidity patterns (16).

Given the above evidence gaps, an in-depth, up-to-date, longitudinal study on multimorbidity patterns and their dynamic evolution among older people in China is crucial. This study aims to systematically explore the co-occurrence mechanisms of various chronic diseases and their potential risk factors, while uncovering the temporal trends of multimorbidity patterns. The findings will provide a more precise foundation for personalized health management and policy development for older people, ultimately alleviating the burden of multimorbidity on both older people and the healthcare system and promoting healthy aging.

## 2 Methods

### 2.1 Data source

Data for this study come from the latest five waves of CHARLS, a nationally representative longitudinal survey of individuals aged 45 years and older in China. High-quality data were collected through face-to-face interviews, structured questionnaires, physical measurements, and blood samples to assess various dimensions of community-level quality of life, socioeconomic status, and health, with a goal to help address the challenges of rapid population aging in China. The baseline survey began in 2011, covering 150 counties, 450 villages, and around 17,708 participants from 10,000 households (3). Follow-up surveys were conducted in 2013, 2015, 2018, and 2020. The most recent publicly available data, from 2020, includes information related to the COVID-19 pandemic and covers 19,300 participants nationwide. This study was approved by the Peking University Biomedical Ethics Committee (IRB00001052-11015) and all participants provided informed consent.

**Figure 1:**
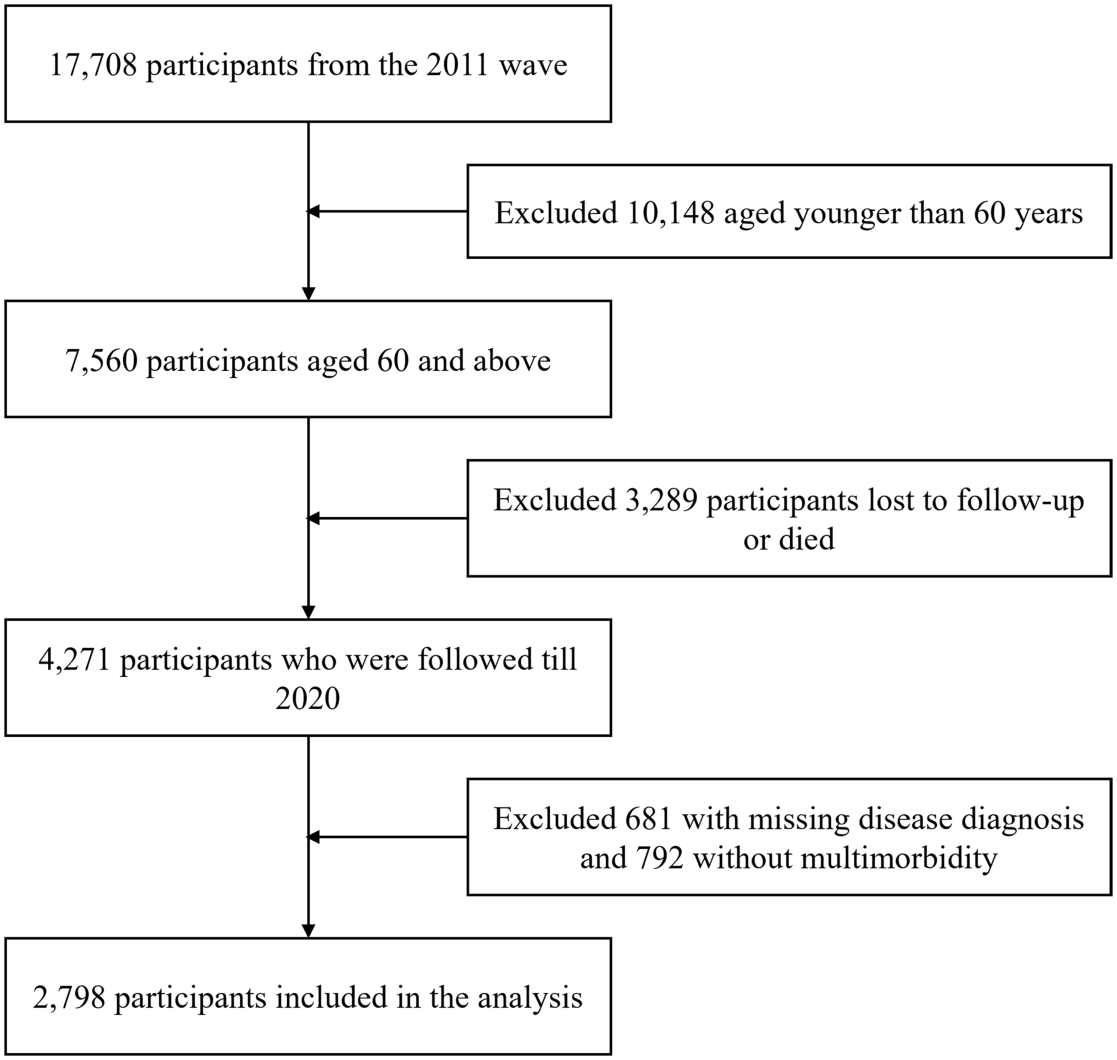
Flowchart for selecting the study sample from the original sample population.

This study was limited to individuals aged 60 and above with at least two chronic conditions who participated in all five waves. Exclusion criteria were: (1) age under 60 at baseline (*N* = 10,148); (2) missing follow-up data or deceased (*N* = 3,289); (3) missing chronic disease data (*N* = 681); (4) having only one chronic condition across all waves (*N* = 792). A total of 2,798 participants were included in the final analysis (Figure 1).

### 2.2 Measures

#### 2.2.1 Chronic conditions and multimorbidity

Multimorbidity was measured using 14 self-reported chronic conditions from CHARLS, including hypertension, dyslipidemia, diabetes, cancer, chronic lung diseases (e.g., chronic bronchitis, emphysema), liver diseases, heart diseases (e.g., myocardial infarction, coronary heart disease, angina), stroke, kidney disease, stomach disease, mental disorders, memory-related diseases (including dementia, brain atrophy, and Parkinson’s disease), arthritis, and asthma. To ensure comprehensive analysis and consistency with previous literature, all 14 conditions were included in the study (3). Each condition was coded as a binary variable (presence = 1). Multimorbidity was defined as the presence of at least two of the 14 chronic conditions in a single individual (22).

#### 2.2.2 Potential influencing factors

Guided by previous literature (23, 24), potential influencing factors were categorized into four groups. Demographic factors included sex (male, female), region (eastern, central, western), residence (urban, rural), age (60–69, 70–79, ≥80 years), BMI (classified by Chinese standards (25): <18.5 kg/m^2^ as underweight, 18.5–23.9 kg/m^2^ as normal, 24–27.9 kg/m^2^ as overweight, ≥28 kg/m^2^ as obese), education level (no education, primary, secondary, vocational, university and above), marital status (single, married/cohabiting), occupation (farmer, non-farmer, unemployed/retired), per capita household income quartiles (low: <1649.75 yuan, lower-middle: 1649.75–5605.83 yuan, upper-middle: 5605.83–20308.33 yuan, high: ≥20308.33 yuan), and health insurance type (employee, resident, other).

Lifestyle and health factors included self-rated health status (good, fair, poor), body pain (none, some, moderate), sleep duration (<4 h, 4–6 h, 6–8 h, ≥8 h), physical activity (highest intensity of weekly activity: vigorous, moderate, light, none), social participation (yes, no), smoking status (yes, no), alcohol consumption (yes, no), functional status [measured using the Activities of Daily Living (ADL) scale and categorized as normal, mildly impaired, moderately impaired, severely impaired], depression (measured using the Center for Epidemiologic Studies Depression [CES-D] scale, with a score of ≥10 indicating depressive symptoms), and cognitive function [measured using the Mini-Mental State Examination (MMSE), where an appropriate score for educational level indicates normal cognition, otherwise cognitive impairment (26)], and life satisfaction (satisfied, fair, dissatisfied).

Factors related to healthcare utilization included outpatient service use (outpatient treatment received in the past month: yes, no), number of outpatient visits per month, monthly out-of-pocket expenses for outpatient (OOPE, after reimbursement), annual out-of-pocket expenses for inpatient (OOPE, after reimbursement), inpatient service use (hospitalization in the past year: yes, no), number of inpatient visits per year, and unmet medical needs during the COVID-19 pandemic (whether participants experienced delays or were unable to seek medical care due to the pandemic: yes, no).

Factors related to community service utilization included the type of community-based care services for older people received by participants (care centers, regular check-ups, home visits, home care beds, community nursing, health management, recreational activities, none), satisfaction with local healthcare (satisfied, fair, dissatisfied), and whether participants had contracted paid family doctor services (yes, no).

### 2.3 Statistical analysis

Descriptive statistics were used to compare the differences in potential influencing factors across different multimorbidity patterns. Categorical variables were reported as frequencies (percentages). Continuous variables were presented as means ± standard deviations (SD). Baseline characteristics across multimorbidity groups were compared using chi-square tests for categorical variables and one-way ANOVA for continuous variables.

Given the observation that the prevalence of most chronic diseases tends to increase over time, this suggests that illness probability among older people varies based on specific chronic conditions and time (Supplementary Figure 1). Therefore, latent class analysis (LCA) was used to cluster chronic diseases into homogenous groups at each time point (27). LCA is able to assign individuals to distinct multimorbidity patterns based on posterior probabilities, offering more objectivity than traditional clustering methods (16, 28). In this model, the latent class membership—i.e., the multimorbidity pattern—is the model-derived outcome, inferred from 14 chronic conditions treated as binary manifest indicators. As the overall distribution and trends of chronic diseases remained relatively stable over the survey period, we used the most recent 2020 data to determine multimorbidity patterns to ensure the timeliness of the analysis. To ensure the robustness of the LCA results, sensitivity analyses were conducted using cross-sectional data from 2011, 2013, 2015, and 2018, with consistent chronic disease variables in each wave.

To further validate the quality of the selected latent class solution, we conducted an external validation using a K-nearest neighbors (KNN) classification approach based on the 2020 wave of data. In this analysis, the original binary indicators of the 14 chronic diseases were used as predictor variables, and the LCA-derived class assignments from 2020 served as the reference outcome. Since LCA is an unsupervised method, we selected KNN—a supervised machine learning algorithm with demonstrated effectiveness in classification tasks—to assess whether the data-driven clusters from LCA could be reliably reproduced (29, 30). This provided an independent evaluation of the coherence and discriminability of the identified multimorbidity patterns (30). The dataset was randomly split into 70% training and 30% testing sets. 10-fold cross-validation was conducted within the training set, and the optimal hyperparameter values were selected to improve model performance. After selecting the optimal model, we used LCA results from five waves of data to construct transition frequency matrices, analyzing pattern transitions for each individual over time. By fitting these matrices, we explored the trajectory of multimorbidity patterns and assessed changes at five time points, providing a comprehensive understanding of their dynamic evolution. All analyses were conducted using Mplus 8.3 and IBM SPSS 26.0, with two-sided *p*-values below 0.05 considered statistically significant.

## 3 Results

### 3.1 Sample characteristics

A total of 2,798 participants aged 60 and above were included in the study (Table 1). The majority were female (53.22%), lived in rural areas (66.51%), were aged 70–79 (61.83%), had primary education or below (81.88%), were mostly unemployed/retired (58.76%), and had basic medical insurance (82.81%). Regarding health status, most had 4–6 h of sleep per night (35.88%), engaged in light physical activity weekly (31.74%), did not participate in social activities (57.04%), did not smoke (55.15%), or drink alcohol (70.94%). Regarding healthcare utilization, the majority had not used outpatient services in the past month (81.52%) or been hospitalized in the past year (75.02%), and 10.36% experienced unmet medical needs during the pandemic. Regarding community services, the majority did not receive any community-based care (73.34%) or have contracted paid family doctor services (94.50%).

**Table 1:** Sample characteristics (*n* = 2,798).

Variables	Total (%)
Sex, *n* (%)	
Female	1,489 (53.22%)
Male	1,309 (46.78%)
Region, *n* (%)	
Eastern	1,032 (36.90%)
Central	858 (30.70%)
Western	908 (32.50%)
Residence, *n* (%)	
Urban	937 (33.49%)
Rural	1861 (66.51%)
Age, *n* (%)	
60–69	593 (21.19%)
70–79	1730 (61.83%)
≥80	475 (16.98%)
BMI (kg/m^2^), *n* (%)	
Underweight	252 (9.01%)
Normal	1,357 (48.50%)
Overweight	830 (29.66%)
Obese	359 (12.83%)
Education level, *n* (%)	
No education	941 (33.63%)
Primary	1,350 (48.25%)
Secondary	399 (14.26%)
Vocational	92 (3.29%)
University and above	16 (0.57%)
Marital status, *n* (%)	
Single	878 (31.38%)
Married/cohabiting	1920 (68.62%)
Occupation, *n* (%)	
Farmer	1,071 (38.28%)
Non-farmer	83 (2.97%)
Unemployed/retired	1,644 (58.76%)
Household income, *n* (%)	
Low	697 (24.91%)
Lower-middle	702 (25.09%)
Upper-middle	700 (25.02%)
High	699 (24.98%)
Health insurance, *n* (%)	
Employee insurance	387 (13.83%)
Resident insurance	2,317 (82.81%)
Other insurance	94 (3.36%)
Self-rated health status, *n* (%)	
Good	400 (14.30%)
Fair	1,330 (47.53%)
Poor	1,068 (38.17%)
Body pain, *n* (%)	
None	960 (34.31%)
Some	1,074 (38.38%)
Moderate	764 (27.31%)
Sleep duration, *n* (%)	
<4 h	760 (27.16%)
4–6 h	1,004 (35.88%)
6–8 h	721 (25.77%)
≥8 h	313 (11.19%)
Physical activity, *n* (%)	
Vigorous activity	659 (23.55%)
Moderate activity	722 (25.80%)
Light activity	888 (31.74%)
No activity	529 (18.91%)
Social participation, *n* (%)	
Yes	1,202 (42.96%)
No	1,596 (57.04%)
Smoking status, *n* (%)	
Yes	1,255 (44.85%)
No	1,543 (55.15%)
Alcohol consumption, *n* (%)	
Yes	813 (29.06%)
No	1985 (70.94%)
Functional status, *n* (%)	
Normal	1705 (60.94%)
Mildly impaired	704 (25.16%)
Moderately impaired	226 (8.08%)
Severely impaired	163 (5.83%)
Depression, *n* (%)	
Yes	1,321 (47.21%)
No	1,477 (52.79%)
Cognitive function, *n* (%)	
Normal cognition	1870 (66.83%)
Cognitive impairment	928 (33.17%)
Life satisfaction, *n* (%)	
Satisfied	1,054 (37.67%)
Fair	1,424 (50.89%)
Dissatisfied	320 (11.44%)
Outpatient service use, *n* (%)	
Yes	517 (18.48%)
No	2,281 (81.52%)
Number of outpatient visits, mean ± SD	0.41 ± 1.36
OOPE for outpatient (yuan), mean ± SD	180.38 ± 1232.56
Inpatient service use, *n* (%)	
Yes	699 (24.98%)
No	2099 (75.02%)
Number of inpatient visits, mean ± SD	0.41 ± 0.92
OOPE for inpatient (yuan), mean ± SD	2033.53 ± 9137.33
Unmet medical needs during COVID-19, *n* (%)	
Yes	290 (10.36%)
No	2,508 (89.64%)
Community-based older adult care services, *n* (%)	
Care centers	19 (0.68%)
Regular check-ups	646 (23.09%)
Home visits	124 (4.43%)
Home care beds	5 (0.18%)
Community nursing	19 (0.68%)
Health management	62 (2.22%)
Recreational activities	66 (2.36%)
None	2052 (73.34%)
Paid family doctor services, *n* (%)	
Yes	154 (5.50%)
No	2,644 (94.50%)
Satisfaction with local healthcare, *n* (%)	
Satisfied	1,212 (43.32%)
Fair	1,125 (40.21%)
Dissatisfied	461 (16.48%)

OOPE, Out-of-Pocket Expenses.

### 3.2 Prevalence and patterns of multimorbidity

**Figure 2:**
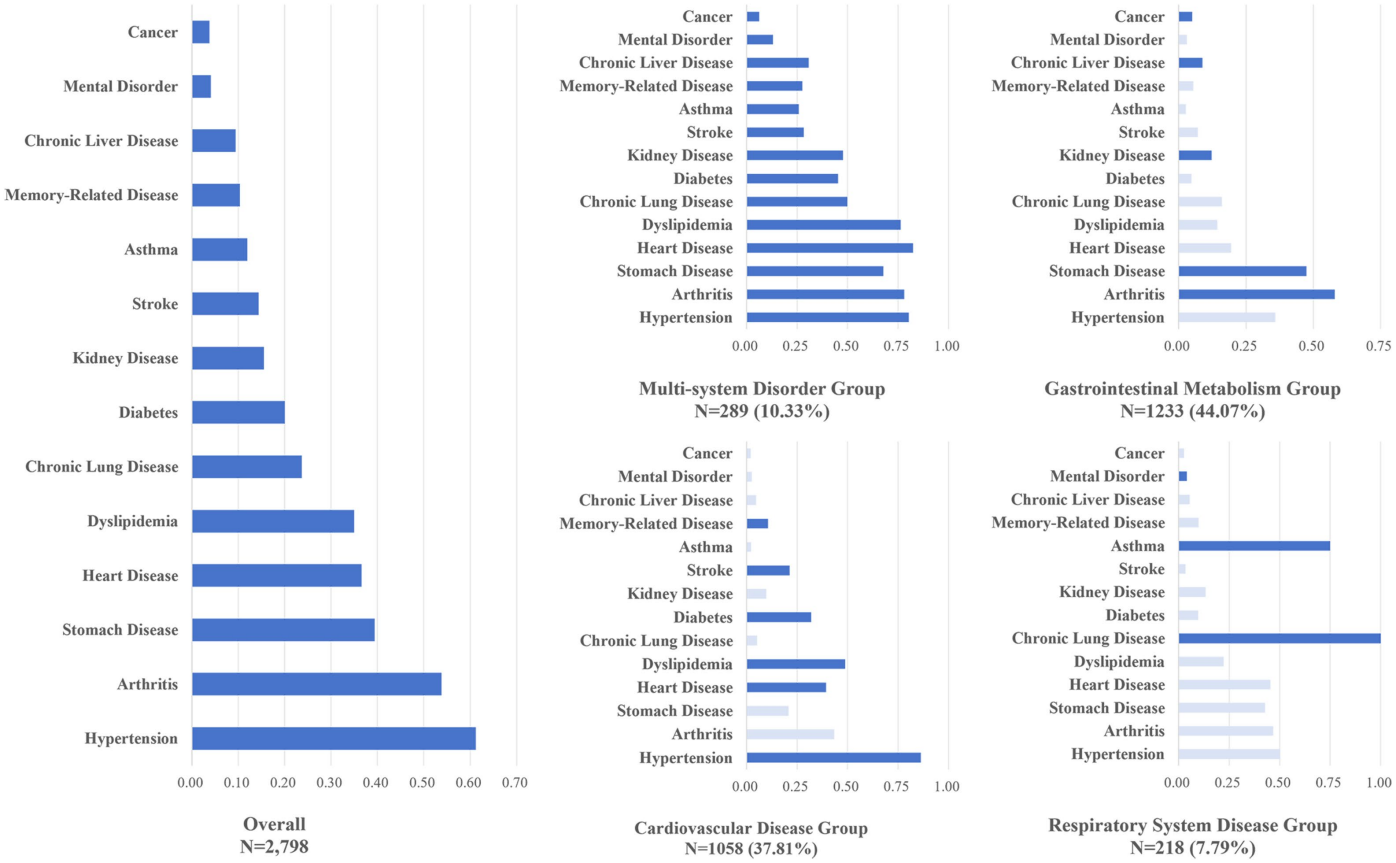
Prevalence of chronic conditions by latent class. Dark blue generally indicates conditions with prevalence higher than the population average.

We evaluated models with 2 to 7 classes using model fit indices and clinical relevance criteria, and found that the four-class model consistently provided the best fit across all five waves, with strong clinical interpretability (detailed fit statistics are provided in Supplementary Tables 2, 3). In addition, external validation using a K-nearest neighbors (KNN) model showed high classification accuracy (96.4%) and strong agreement (Kappa = 0.944), further supporting the robustness of the identified latent class structure (Supplementary Table 4). Based on the probabilities of 14 chronic conditions and overall prevalence, the four patterns were named: multi-system disorder group (*N* = 289, 10.33%); gastrointestinal metabolism group (*N* = 1,233, 44.07%); cardiovascular disease group (*N* = 1,058, 37.81%); and respiratory disease group (*N* = 218, 7.79%) (Figure 2). The multi-system disorder group had high prevalence across all conditions, while the gastrointestinal metabolism group was marked by stomach, kidney, liver diseases, and arthritis. The cardiovascular disease group included hypertension, heart disease, dyslipidemia, diabetes, and stroke, and the respiratory disease group had high rates of chronic lung disease and asthma.

In addition, comparisons of demographic and health-related characteristics highlighted clear heterogeneity across the four multimorbidity groups (Supplementary Table 1). The multi-system disorders group showed the highest burden of physical limitations. The gastrointestinal metabolism group had the largest proportion of rural residents and individuals with lower educational attainment. The cardiovascular disease group was more urbanized, with a greater number of overweight or obese individuals. The respiratory disease group had the highest prevalence of smoking.

### 3.3 Trajectory of multimorbidity patterns

**Figure 3:**
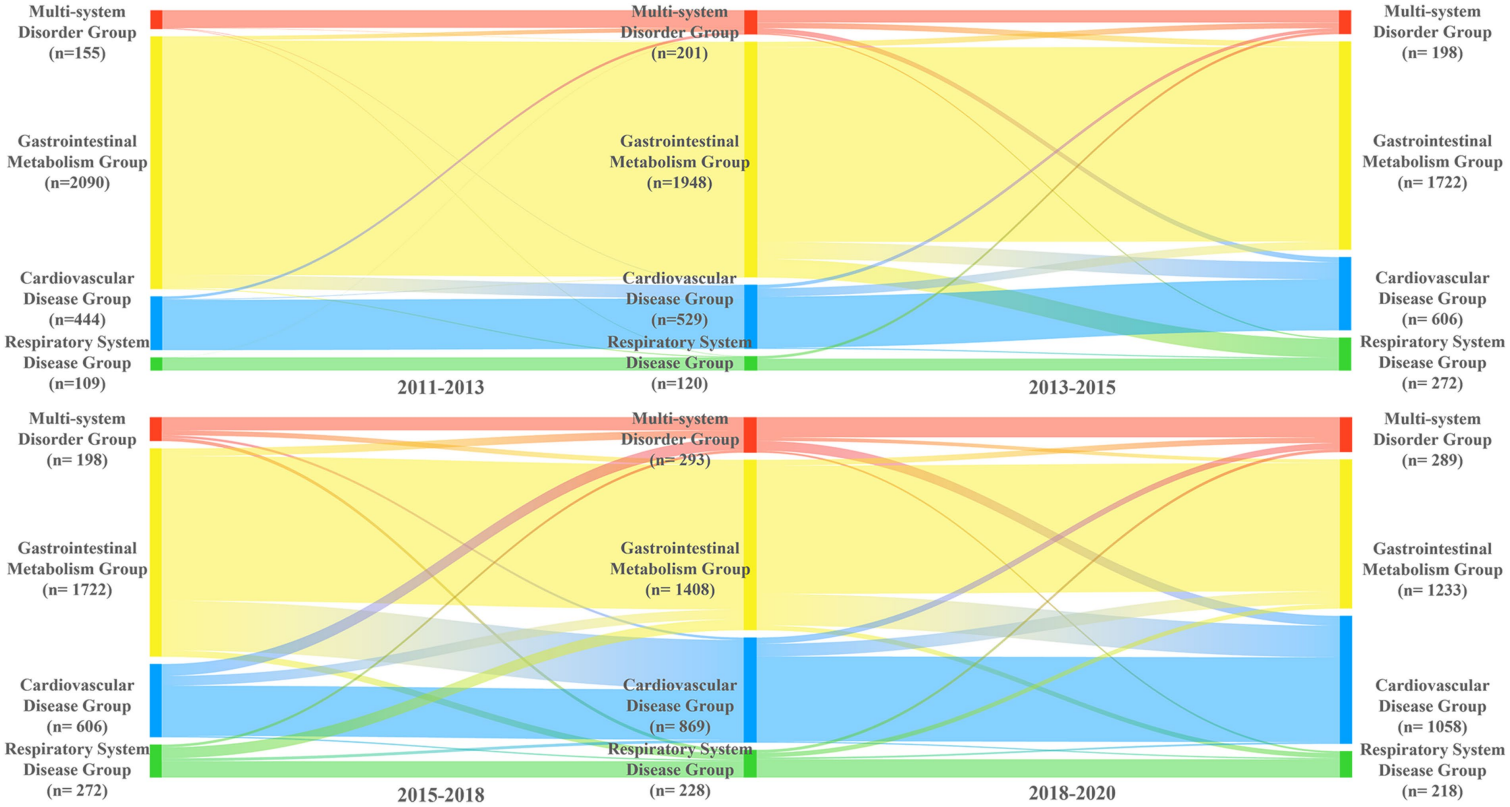
Trajectory of multimorbidity patterns among older chinese people across five time points.

Based on LCA results from five waves, we identified the modal class assignment for each multimorbidity pattern at each time point, forming an initial probability matrix and transition frequency matrices between consecutive waves (Supplementary Table 5). Figure 3 shows the trajectory of multimorbidity patterns among older people in China. From 2011 to 2020, the gastrointestinal metabolism group decreased (74.70%, *N* = 2,090, to 44.07%, *N* = 1,233), the cardiovascular disease group increased (15.87%, *N* = 444, to 37.81%, *N* = 1,058), the multi-system disorder group increased (5.54%, *N* = 155, to 10.33%, *N* = 289), and the respiratory disease group increased (3.90%, N = 109, to 7.79%, *N* = 218).

From 2011 to 2013, most individuals remained in their original multimorbidity patterns, with self-transition rates above 90%. However, pattern stability declined over time, recovering only between 2018 and 2020. The gastrointestinal metabolism group was the least stable, with retention dropping from 92.78% from 2011 to 2013 to 75.28% from 2018 to 2020. Many individuals transitioned from the gastrointestinal metabolism group to others, becoming the main source of shifts into the cardiovascular disease group (7.29% from 2013 to 2015, 23.81% from 2015 to 2018, 18.54% from 2018 to 2020). The multi-system disorder group remained relatively stable, with a retention rate of around 50.00% from 2013 to 2020. The stability of the respiratory disease group dropped to 49.63% during 2015–2018, with 33.46% of individuals shifting to the gastrointestinal metabolism group.

## 4 Discussion

This study used the latest five waves of CHARLS to analyze multimorbidity patterns and their trajectories among older people in China from 2011 to 2020. To our knowledge, this is the first and most extensive national study utilizing large-scale, long-term longitudinal data to assess multimorbidity patterns and their dynamic changes over the past decade in China. We identified four main patterns: multi-system disorders; gastrointestinal metabolism; cardiovascular diseases; and respiratory diseases. There were significant differences in prevalence and transition of these patterns, with stability declining between 2013 and 2018, especially in the gastrointestinal metabolism group whereby individuals shifted to the cardiovascular disease group. Stability improved from 2018 to 2020.

Most participants in our sample were women, potentially due to postmenopausal hormonal changes that increase chronic disease risk (3). The prevalence of multimorbidity rises with age but declines slightly after 80, possibly due to fewer chronic conditions among older people or recall bias (31). Low education levels were common, which are linked to higher disease risk, while higher education helps maintain cognitive function and slow disease progression (32, 33). Most participants lived in rural areas with low social activity, though social participation can improve physical and mental health in older adults, and urban residents tend to be healthier (7). Sleeping less than 6 h was also found to increase multimorbidity risk, highlighting the importance of sleep (34).

Low utilization of outpatient and inpatient services can delay the diagnosis and management of chronic diseases, increasing multimorbidity risk. However, studies show multimorbid patients often have higher healthcare utilization (22). This discrepancy may stem from most of our participants living in rural areas, where healthcare accessibility and utilization are relatively low, thus affecting disease management (3, 35). Some participants reported unmet medical needs during the COVID-19 pandemic, consistent with prior research highlighting the disruption of chronic disease management and the increased complexity of long-term conditions during public health crises (36, 37). Additionally, most participants lacked community services and family doctor support, despite evidence that family doctors improve chronic disease management and service utilization (38). This suggests the need for better integration of community healthcare services, particularly through family doctor programs.

We identified four multimorbidity patterns among older people in China. The multi-system disorder group had high prevalence rates for all 14 chronic conditions. These coexisting conditions were accompanied by substantial functional limitations and pain burden, contributing to elevated healthcare utilization and costs and indicating an increased demand for medical and supportive care (39, 40). The gastrointestinal metabolism group was the most prevalent multimorbidity pattern over the past decade, with 44.07% in this group; multimorbidity in this group may be due to gastrointestinal side effects from use of nonsteroidal anti-inflammatory drugs (NSAIDs) in arthritis patients, leading to the high co-occurrence of digestive disorders and arthritis (41, 42). Additionally, liver disease that increases the risk of chronic kidney disease may also contribute to the coexistence of these conditions (43). Patients with gastrointestinal disorders tend to have higher healthcare demands, a burden potentially compounded by the group’s high proportion of rural residents and individuals with lower educational attainment (20). Our findings highlight the significance of this pattern, though it has not been found in other studies, possibly because gastrointestinal disorders were not included in previous research (16).

The cardiovascular disease group, characterized by a higher proportion of overweight individuals and urban residents, also showed elevated risks of hospitalization and organ failure (20, 44). Given their need for long-term treatment and the substantial healthcare costs involved, cardiovascular diseases represent a major contributor to the global economic burden of chronic conditions (40, 45). Therefore, better disease management and medical support strategies are essential for patients with cardiovascular diseases. The respiratory disease group, comprising chronic lung and asthma conditions, involves conditions with shared etiologies and a high risk of deterioration, leading to elevated healthcare utilization (13).

Our analysis of the multimorbidity trajectory over a decade showed a sharp decline in the gastrointestinal metabolism group, from 74.70% in 2011 to 44.07% in 2020, and a significant rise in the cardiovascular group, from 15.87% in 2011 to 37.81% in 2020. Further analysis revealed that the gastrointestinal metabolism group was the least stable, with many individuals transitioning to the cardiovascular group, suggesting gastrointestinal issues may be an early indicator of cardiovascular disease development. Studies have linked gastrointestinal conditions with an elevated risk of cardiovascular disease through mechanisms like metabolic abnormalities, inflammation, and oxidative stress (46, 47). Additionally, liver and kidney diseases, among other metabolic conditions, are likely important risk factors contributing to cardiovascular disease progression (46, 48). Therefore, early screening and integrated management of cardiovascular risk are crucial for patients with gastrointestinal and metabolic disorders to prevent disease progression.

The shift from high prevalence of gastrointestinal and metabolic disorders to cardiovascular diseases over the study period aligns with other studies (20, 49). Population aging, lifestyle changes, and environmental influences likely contribute to this trend. For example, high-salt and high-fat diets, physical inactivity, and increased stress may accelerate cardiovascular disease development (50). Cardiovascular diseases, now a leading global health issue, are the top cause of death among older people in China (8, 51). Yet, awareness, treatment, and control rates for cardiovascular diseases remain insufficient (51). Public health policies should prioritize cardiovascular disease prevention and management, focusing on health education, lifestyle interventions, and early screening. The rising proportion of the multi-system disorder group further indicates that as age increases, the likelihood of multiple chronic conditions also rises, leading to greater complexity in disease management (28). The dynamic changes in the respiratory disease group are likely influenced by factors such as environmental pollution, smoking, and occupational exposure (52).

### 4.1 Strengths and limitations

Strengths include use of the latest nationally representative longitudinal data among older people in China. We thoroughly examined the co-occurrence mechanisms and risk factors of chronic diseases across five data waves, systematically analyzing long-term dynamic changes and evolution in multimorbidity patterns. Unlike previous studies, our decade-long follow-up provides comprehensive evidence, filling research gaps and offering guidance for targeted public health policies and personalized health management, particularly for managing chronic diseases in aging populations.

However, this study has limitations. First, self-reported data may introduce information and recall bias. Second, only 14 chronic diseases were included, potentially omitting other conditions that affect older adults’ health. Third, future research should explore the risk factors influencing multimorbidity transitions and their mechanisms, including potential demographic or behavioral contributors, and consider incorporating a broader range of clinically or symptom-based conditions to better capture the complexity of multimorbidity in older adults.

## 5 Conclusion

This study used five waves of CHARLS data to analyze multimorbidity patterns and their evolution among older people in China from 2011 to 2020, identifying four primary patterns: multi-system disorders; gastrointestinal metabolism; cardiovascular diseases; and respiratory diseases. The findings show a shift in disease burden from gastrointestinal and metabolic disorders to cardiovascular diseases, with gastrointestinal issues potentially serving as early indicators of cardiovascular conditions. These insights can improve prevention, intervention, and treatment strategies, optimize healthcare resource allocation, and carry significant policy and clinical implications.

## Data Availability

The datasets presented in this study can be found in online repositories. The names of the repository/repositories and accession number(s) can be found at: http://charls.Pku.edu.cn/en.
