# Supplementary material for: Identification and trajectory of multimorbidity patterns among older people in China: a longitudinal study based on the China health and retirement longitudinal study 2011–2020 data
Source: Front Public Health. 2025 Jul 21;13:1597224. doi: 10.3389/fpubh.2025.1597224 (PMC12318766; doi:10.3389/fpubh.2025.1597224)
Supplement: Supplementary file 1 [file Data_Sheet_1.docx]

Supplementary Material

Supplementary Table 1 Sample characteristics (n = 2,798)

Supplementary Figure 1 Prevalence of 14 Chronic Diseases Among the Older Adult (2011-2020).

Supplementary Table 2 Comparison of Fit Statistics for LCA Models.

Supplementary Table 3 Latent Class Assignment Probabilities from the 2020 LCA Model.

Supplementary Table 4 Confusion matrix and performance metrics of KNN classification model based on 2020 data

Supplementary Table 5 Initial Probability Matrix and Transition Frequency Matrix for Four Multimorbidity Patterns.

Supplementary Table 1 Sample characteristics (n = 2,798)

| Variables | Total (%) | Multi-system disorders group（10.33%) | Gastrointestinal metabolism group（44.07%) | Cardiovascular disease group（37.81%) | Respiratory disease group  （7.79%) | *P* |
| --- | --- | --- | --- | --- | --- | --- |
| Sex, n (%)^a^ |  |  |  |  |  | **<0.001** |
| Female | 1489 (53.22%) | 150 (51.90%) | 660 (53.53%) | 600 (56.71%) | 79 (36.24%) |  |
| Male | 1309 (46.78%) | 139 (48.10%) | 573 (46.47%) | 458 (43.29%) | 139 (63.76%) |  |
| Region, n (%)^a^ |  |  |  |  |  | **<0.001** |
| Eastern | 1032 (36.90%) | 97 (33.56%) | 417 (33.82%) | 454 (42.91%) | 64 (29.36%) |  |
| Central | 858 (30.70%) | 88 (30.45%) | 352 (28.55%) | 337 (31.85%) | 81 (37.16%) |  |
| Western | 908 (32.50%) | 104 (35.99%) | 464 (37.63%) | 267 (25.24%) | 73 (33.49%) |  |
| Residence, n (%)^a^ |  |  |  |  |  | **0.001** |
| Urban | 937 (33.49%) | 96 (33.22%) | 368 (29.85%) | 400 (37.81%) | 73 (33.49%) |  |
| Rural | 1861 (66.51%) | 193 (66.78%) | 865 (70.15%) | 658 (62.19%) | 145 (66.51%) |  |
| Age, n (%)^a^ |  |  |  |  |  | **0.010** |
| 60-69 | 593 (21.19%) | 66 (22.84%) | 287 (23.28%) | 209 (19.75%) | 31 (14.22%) |  |
| 70-79 | 1730 (61.83%) | 186 (64.36%) | 725 (58.80%) | 674 (63.71%) | 145 (66.51%) |  |
| ≥80 | 475 (16.98%) | 37 (12.80%) | 221 (17.92%) | 175 (16.54%) | 42 (19.27%) |  |
| BMI (kg/m^2^), n (%)^a^ |  |  |  |  |  | **<0.001** |
| Underweight | 252 (9.01%) | 17 (5.88%) | 143 (11.60%) | 56 (5.29%) | 36 (16.51%) |  |
| Normal | 1357 (48.50%) | 131 (45.33%) | 699 (56.69%) | 406 (38.37%) | 121 (55.50%) |  |
| Overweight | 830 (29.66%) | 74 (25.61%) | 294 (23.84%) | 413 (39.04%) | 49 (22.48%) |  |
| Obese | 359 (12.83%) | 67 (23.18%) | 97 (7.87%) | 183 (17.30%) | 12 (5.50%) |  |
| Education level, n (%)^a^ |  |  |  |  |  | **<0.001** |
| No education | 941 (33.63%) | 77 (26.64%) | 465 (37.71%) | 348 (32.89%) | 51 (23.39%) |  |
| Primary | 1350 (48.25%) | 142 (49.13%) | 586 (47.53%) | 500 (47.26%) | 122 (55.96%) |  |
| Secondary | 399 (14.26%) | 58 (20.07%) | 140 (11.35%) | 164 (15.50%) | 37 (16.97%) |  |
| Vocational | 92 (3.29%) | 11 (3.81%) | 36 (2.92%) | 39 (3.69%) | 6 (2.75%) |  |
| University and above | 16 (0.57%) | 1 (0.35%) | 6 (0.49%) | 7 (0.66%) | 2 (0.92%) |  |
| Marital status, n (%)^a^ |  |  |  |  |  | 0.948 |
| Single | 878 (31.38%) | 94 (32.53%) | 390 (31.63%) | 327 (30.91%) | 67 (30.73%) |  |
| Married/cohabiting | 1920 (68.62%) | 195 (67.47%) | 843 (68.37%) | 731 (69.09%) | 151 (69.27%) |  |
| Occupation, n (%)^a^ |  |  |  |  |  | **<0.001** |
| Farmer | 1071 (38.28%) | 92 (31.83%) | 568 (46.07%) | 350 (33.08%) | 61 (27.98%) |  |
| Non-farmer | 83 (2.97%) | 4 (1.38%) | 47 (3.81%) | 27 (2.55%) | 5 (2.29%) |  |
| Unemployed/retired | 1644 (58.76%) | 193 (66.78%) | 618 (50.12%) | 681 (64.37%) | 152 (69.72%) |  |
| Household income, n (%)^a^ |  |  |  |  |  | **<0.001** |
| Low | 697 (24.91%) | 61 (21.11%) | 327 (26.52%) | 252 (23.82%) | 57 (26.15%) |  |
| Lower-middle | 702 (25.09%) | 62 (21.45%) | 341 (27.66%) | 232 (21.93%) | 67 (30.73%) |  |
| Upper-middle | 700 (25.02%) | 73 (25.26%) | 302 (24.49%) | 282 (26.65%) | 43 (19.72%) |  |
| High | 699 (24.98%) | 93 (32.18%) | 263 (21.33%) | 292 (27.60%) | 51 (23.39%) |  |
| Health insurance, n (%)^a^ |  |  |  |  |  | **<0.001** |
| Employee insurance | 387 (13.83%) | 52 (17.99%) | 123 (9.98%) | 185 (17.49%) | 27 (12.39%) |  |
| Resident insurance | 2317 (82.81%) | 227 (78.55%) | 1070 (86.78%) | 835 (78.92%) | 185 (84.86%) |  |
| Other insurance | 94 (3.36%) | 10 (3.46%) | 40 (3.24%) | 38 (3.59%) | 6 (2.75%) |  |
| Self-rated health status, n (%)^a^ |  |  |  |  |  | **<0.001** |
| Good | 400 (14.30%) | 21 (7.27%) | 189 (15.33%) | 165 (15.60%) | 25 (11.47%) |  |
| Fair | 1330 (47.53%) | 111 (38.41%) | 635 (51.50%) | 500 (47.26%) | 84 (38.53%) |  |
| Poor | 1068 (38.17%) | 157 (54.33%) | 409 (33.17%) | 393 (37.15%) | 109 (50.00%) |  |
| Body pain, n (%)^a^ |  |  |  |  |  | **<0.001** |
| None | 960 (34.31%) | 43 (14.88%) | 416 (33.74%) | 429 (40.55%) | 72 (33.03%) |  |
| Some | 1074 (38.38%) | 103 (35.64%) | 513 (41.61%) | 377 (35.63%) | 81 (37.16%) |  |
| Moderate | 764 (27.31%) | 143 (49.48%) | 304 (24.66%) | 252 (23.82%) | 65 (29.82%) |  |
| Sleep duration, n (%)^a^ |  |  |  |  |  | **0.001** |
| <4 hours | 760 (27.16%) | 99 (34.26%) | 353 (28.63%) | 246 (23.25%) | 62 (28.44%) |  |
| 4-6 hours | 1004 (35.88%) | 111 (38.41%) | 431 (34.96%) | 384 (36.29%) | 78 (35.78%) |  |
| 6-8 hours | 721 (25.77%) | 61 (21.11%) | 310 (25.14%) | 301 (28.45%) | 49 (22.48%) |  |
| ≥8 hours | 313 (11.19%) | 18 (6.23%) | 139 (11.27%) | 127 (12.00%) | 29 (13.30%) |  |
| Physical activity, n (%)^a^ |  |  |  |  |  | **<0.001** |
| Vigorous activity | 659 (23.55%) | 66 (22.84%) | 371 (30.09%) | 184 (17.39%) | 38 (17.43%) |  |
| Moderate activity | 722 (25.80%) | 79 (27.34%) | 324 (26.28%) | 274 (25.90%) | 45 (20.64%) |  |
| Light activity | 888 (31.74%) | 98 (33.91%) | 343 (27.82%) | 363 (34.31%) | 84 (38.53%) |  |
| No activity | 529 (18.91%) | 46 (15.92%) | 195 (15.82%) | 237 (22.40%) | 51 (23.39%) |  |
| Social participation, n (%)^a^ |  |  |  |  |  | 0.664 |
| Yes | 1202 (42.96%) | 134 (46.37%) | 522 (42.34%) | 453 (42.82%) | 93 (42.66%) |  |
| No | 1596 (57.04%) | 155 (53.63%) | 711 (57.66%) | 605 (57.18%) | 125 (57.34%) |  |
| Smoking status, n (%)^a^ |  |  |  |  |  | **<0.001** |
| Yes | 1255 (44.85%) | 145 (50.17%) | 557 (45.17%) | 410 (38.75%) | 143 (65.60%) |  |
| No | 1543 (55.15%) | 144 (49.83%) | 676 (54.83%) | 648 (61.25%) | 75 (34.40%) |  |
| Alcohol consumption, n (%)^a^ |  |  |  |  |  | **0.005** |
| Yes | 813 (29.06%) | 84 (29.07%) | 397 (32.20%) | 269 (25.43%) | 63 (28.90%) |  |
| No | 1985 (70.94%) | 205 (70.93%) | 836 (67.80%) | 789 (74.57%) | 155 (71.10%) |  |
| Functional status, n (%)^a^ |  |  |  |  |  | **<0.001** |
| Normal | 1705 (60.94%) | 139 (48.10%) | 806 (65.37%) | 638 (60.30%) | 122 (55.96%) |  |
| Mildly impaired | 704 (25.16%) | 83 (28.72%) | 299 (24.25%) | 253 (23.91%) | 69 (31.65%) |  |
| Moderately impaired | 226 (8.08%) | 41 (14.19%) | 82 (6.65%) | 90 (8.51%) | 13 (5.96%) |  |
| Severely impaired | 163 (5.83%) | 26 (9.00%) | 46 (3.73%) | 77 (7.28%) | 14 (6.42%) |  |
| Depression, n (%)^a^ |  |  |  |  |  | **<0.001** |
| Yes | 1321 (47.21%) | 180 (62.28%) | 562 (45.58%) | 474 (44.80%) | 105 (48.17%) |  |
| No | 1477 (52.79%) | 109 (37.72%) | 671 (54.42%) | 584 (55.20%) | 113 (51.83%) |  |
| Cognitive function, n (%)^a^ |  |  |  |  |  | 0.434 |
| Normal cognition | 1870 (66.83%) | 181 (62.63%) | 826 (66.99%) | 717 (67.77%) | 146 (66.97%) |  |
| Cognitive impairment | 928 (33.17%) | 108 (37.37%) | 407 (33.01%) | 341 (32.23%) | 72 (33.03%) |  |
| Life satisfaction, n (%)^a^ |  |  |  |  |  | **<0.001** |
| Satisfied | 1054 (37.67%) | 80 (27.68%) | 445 (36.09%) | 445 (42.06%) | 84 (38.53%) |  |
| Fair | 1424 (50.89%) | 154 (53.29%) | 653 (52.96%) | 504 (47.64%) | 113 (51.83%) |  |
| Dissatisfied | 320 (11.44%) | 55 (19.03%) | 135 (10.95%) | 109 (10.30%) | 21 (9.63%) |  |
| Outpatient service use, n (%)^a^ |  |  |  |  |  | **<0.001** |
| Yes | 517 (18.48%) | 89 (30.80%) | 210 (17.03%) | 178 (16.82%) | 40 (18.35%) |  |
| No | 2281 (81.52%) | 200 (69.20%) | 1023 (82.97%) | 880 (83.18%) | 178 (81.65%) |  |
| Number of outpatient visits, mean±SD^b^ | 0.41 ± 1.36 | 0.77 ± 1.73 | 0.34 ± 1.23 | 0.39 ± 1.44 | 0.37 ± 1.07 | **<0.001** |
| OOPE for outpatient (yuan), mean±SD^b^ | 180.38 ± 1232.56 | 210.80 ± 690.44 | 210.33 ± 1621.60 | 155.86 ± 895.13 | 89.61 ± 315.21 | 0.475 |
| Inpatient service use, n (%)^a^ |  |  |  |  |  | **<0.001** |
| Yes | 699 (24.98%) | 120 (41.52%) | 238 (19.30%) | 258 (24.39%) | 83 (38.07%) |  |
| No | 2099 (75.02%) | 169 (58.48%) | 995 (80.70%) | 800 (75.61%) | 135 (61.93%) |  |
| Number of inpatient visits, mean±SD^b^ | 0.41 ± 0.92 | 0.84 ± 1.42 | 0.28 ± 0.70 | 0.40 ± 0.92 | 0.61 ± 0.99 | **<0.001** |
| OOPE for inpatient (yuan), mean±SD^b^ | 2033.53 ± 9137.33 | 3919.32 ± 15974.44 | 1541.03 ± 7494.55 | 2035.39 ± 8420.53 | 2310.12 ± 8185.99 | **0.001** |
| Unmet medical needs during COVID-19, n (%)^a^ |  |  |  |  |  | **<0.001** |
| Yes | 290 (10.36%) | 67 (23.18%) | 95 (7.70%) | 95 (8.98%) | 33 (15.14%) |  |
| No | 2508 (89.64%) | 222 (76.82%) | 1138 (92.30%) | 963 (91.02%) | 185 (84.86%) |  |
| Community-based elderly care services, n (%)^a^ |  |  |  |  |  |  |
| Care centers | 19 (0.68%) | 2 (0.69%) | 9 (0.73%) | 7 (0.66%) | 1 (0.46%) | 0.976 |
| Regular check-ups | 646 (23.09%) | 63 (21.80%) | 273 (22.14%) | 256 (24.20%) | 54 (24.77%) | 0.578 |
| Home visits | 124 (4.43%) | 16 (5.54%) | 52 (4.22%) | 45 (4.25%) | 11 (5.05%) | 0.743 |
| Home care beds | 5 (0.18%) | 1 (0.35%) | 2 (0.16%) | 2 (0.19%) | 0 (0.00%) | 0.833 |
| Community nursing | 19 (0.68%) | 4 (1.38%) | 4 (0.32%) | 11 (1.04%) | 0 (0.00%) | **0.047** |
| Health management | 62 (2.22%) | 8 (2.77%) | 28 (2.27%) | 23 (2.17%) | 3 (1.38%) | 0.767 |
| Recreational activities | 66 (2.36%) | 7 (2.42%) | 35 (2.84%) | 22 (2.08%) | 2 (0.92%) | 0.313 |
| None | 2052 (73.34%) | 213 (73.70%) | 909 (73.72%) | 774 (73.16%) | 156 (71.56%) | 0.923 |
| Paid family doctor services, n (%)^a^ |  |  |  |  |  | **0.070** |
| Yes | 154 (5.50%) | 16 (5.54%) | 54 (4.38%) | 73 (6.90%) | 11 (5.05%) |  |
| No | 2644 (94.50%) | 273 (94.46%) | 1179 (95.62%) | 985 (93.10%) | 207 (94.95%) |  |
| Satisfaction with local healthcare, n (%)^a^ |  |  |  |  |  | **0.009** |
| Satisfied | 1212 (43.32%) | 116 (40.14%) | 527 (42.74%) | 476 (44.99%) | 93 (42.66%) |  |
| Fair | 1125 (40.21%) | 105 (36.33%) | 526 (42.66%) | 410 (38.75%) | 84 (38.53%) |  |
| Dissatisfied | 461 (16.48%) | 68 (23.53%) | 180 (14.60%) | 172 (16.26%) | 41 (18.81%) |  |

^a^ Categorical variables were tested using the Pearson chi-square test.

^b^ Continuous variables were tested using one-way analysis of variance (ANOVA).

Supplementary Figure 1 Prevalence of 14 Chronic Diseases Among the Older Adult (2011-2020).

Supplementary Table 2-1 Comparison of Fit Statistics for LCA Models (2011)

| LCA Models | AIC↓ | BIC↓ | aBIC | Entropy↑ | LMR (p) | BLRT (p) | Class Membership Probabilities |
| --- | --- | --- | --- | --- | --- | --- | --- |
| 2-Class | 24860.819 | 25032.982 | 24940.839 | 0.533 | <0.001 | <0.001 | 0.26/0.74 |
| 3-Class | 24594.107 | 24855.320 | 24715.517 | 0.635 | <0.001 | <0.001 | 0.68/0.23/0.09 |
| **4-Class** | **24510.517** | **24860.780** | **24673.317** | **0.701** | **0.019** | **<0.001** | **0.08/0.04/0.19/0.69** |
| 5-Class | 24478.238 | 24917.550 | 24682.427 | 0.582 | 0.305 | <0.001 | 0.11/0.06/0.07/0.37/0.39 |
| 6-Class | 24451.820 | 24980.183 | 24697.400 | 0.625 | 0.585 | <0.001 | 0.08/0.06/0.31/0.09/0.03/0.43 |
| 7-Class | 24447.472 | 25064.885 | 24734.441 | 0.704 | 0.006 | 0.375 | 0.02/0.08/0.50/0.09/0.01/0.25/0.05 |

AIC: Akaike Information Criterion.

BIC: Bayesian Information Criterion.

aBIC: Adjusted Bayesian Information Criterion.

LMR: Lo-Mendell-Rubin Likelihood Ratio Test.

BLRT: Bootstrap Likelihood Ratio Test.

Supplementary Table 2-2 Comparison of Fit Statistics for LCA Models (2013)

| LCA Models | AIC↓ | BIC↓ | aBIC | Entropy↑ | LMR (p) | BLRT (p) | Class Membership Probabilities |
| --- | --- | --- | --- | --- | --- | --- | --- |
| 2-Class | 26745.950 | 26918.113 | 26825.970 | 0.477 | <0.001 | <0.001 | 0.30/0.70 |
| 3-Class | 26472.176 | 26733.389 | 26593.586 | 0.612 | <0.001 | <0.001 | 0.26/0.65/0.09 |
| **4-Class** | **26360.553** | **26710.816** | **26523.353** | **0.658** | **0.006** | **<0.001** | **0.10/0.04/0.62/0.24** |
| 5-Class | 26328.839 | 26768.152 | 26533.029 | 0.587 | 0.684 | <0.001 | 0.10/0.07/0.06/0.36/0.41 |
| 6-Class | 26301.015 | 26829.378 | 26546.595 | 0.595 | 0.604 | <0.001 | 0.06/0.06/0.08/0.09/0.37/0.34 |
| 7-Class | 26295.949 | 26913.362 | 26582.918 | 0.637 | 0.217 | 0.167 | 0.10/0.05/0.03/0.09/0.32/0.01/0.40 |

AIC: Akaike Information Criterion.

BIC: Bayesian Information Criterion.

aBIC: Adjusted Bayesian Information Criterion.

LMR: Lo-Mendell-Rubin Likelihood Ratio Test.

BLRT: Bootstrap Likelihood Ratio Test.

Supplementary Table 2-3 Comparison of Fit Statistics for LCA Models (2015)

| LCA Models | AIC↓ | BIC↓ | aBIC | Entropy↑ | LMR (p) | BLRT (p) | Class Membership Probabilities |
| --- | --- | --- | --- | --- | --- | --- | --- |
| 2-Class | 31114.022 | 31286.185 | 31194.042 | 0.530 | <0.001 | <0.001 | 0.72/0.28 |
| 3-Class | 30730.029 | 30991.243 | 30851.440 | 0.658 | <0.001 | <0.001 | 0.11/0.26/0.63 |
| **4-Class** | **30588.992** | **30939.255** | **30751.792** | **0.650** | **0.027** | **<0.001** | **0.09/0.57/0.24/0.10** |
| 5-Class | 30536.867 | 30976.180 | 30741.057 | 0.620 | 0.479 | <0.001 | 0.11/0.18/0.08/0.18/0.45 |
| 6-Class | 30508.814 | 31037.177 | 30754.394 | 0.623 | 0.132 | <0.001 | 0.03/0.07/0.31/0.09/0.16/0.34 |
| 7-Class | 30491.911 | 31109.323 | 30778.880 | 0.643 | 0.558 | <0.001 | 0.04/0.02/0.30/0.14/0.09/0.35/0.06 |

AIC: Akaike Information Criterion.

BIC: Bayesian Information Criterion.

aBIC: Adjusted Bayesian Information Criterion.

LMR: Lo-Mendell-Rubin Likelihood Ratio Test.

BLRT: Bootstrap Likelihood Ratio Test.

Supplementary Table 2-4 Comparison of Fit Statistics for LCA Models (2018)

| LCA Models | AIC↓ | BIC↓ | aBIC | Entropy↑ | LMR (p) | BLRT (p) | Class Membership Probabilities |
| --- | --- | --- | --- | --- | --- | --- | --- |
| 2-Class | 34764.922 | 34937.086 | 34844.943 | 0.708 | <0.001 | <0.001 | 0.23/0.77 |
| 3-Class | 34318.464 | 34579.677 | 34439.874 | 0.623 | 0.018 | <0.001 | 0.24/0.60/0.16 |
| **4-Class** | **34079.539** | **34429.802** | **34242.339** | **0.615** | **<0.001** | **<0.001** | **0.11/0.10/0.47/0.32** |
| 5-Class | 34012.747 | 34452.059 | 34216.936 | 0.612 | 0.569 | <0.001 | 0.05/0.15/0.11/0.43/0.26 |
| 6-Class | 33986.818 | 34515.181 | 34232.398 | 0.638 | 0.400 | <0.001 | 0.08/0.04/0.37/0.06/0.11/0.34 |
| 7-Class | 33968.989 | 34586.401 | 34255.958 | 0.651 | 0.086 | <0.001 | 0.37/0.04/0.33/0.09/0.08/0.06/0.03 |

AIC: Akaike Information Criterion.

BIC: Bayesian Information Criterion.

aBIC: Adjusted Bayesian Information Criterion.

LMR: Lo-Mendell-Rubin Likelihood Ratio Test.

BLRT: Bootstrap Likelihood Ratio Test.

Supplementary Table 2-5 Comparison of Fit Statistics for LCA Models (2020)

| LCA Models | AIC↓ | BIC↓ | aBIC | Entropy↑ | LMR (p) | BLRT (p) | Class Membership Probabilities |
| --- | --- | --- | --- | --- | --- | --- | --- |
| 2-Class | 36079.633 | 36251.797 | 36159.654 | 0.769 | <0.001 | <0.001 | 0.18/0.82 |
| 3-Class | 35587.663 | 35848.877 | 35709.074 | 0.620 | 0.005 | <0.001 | 0.14/0.36/0.50 |
| **4-Class** | **35312.389** | **35662.652** | **35475.189** | **0.629** | **<0.001** | **<0.001** | **0.42/0.12/0.37/0.09** |
| 5-Class | 35253.890 | 35693.203 | 35458.080 | 0.627 | 0.529 | <0.001 | 0.04/0.09/0.24/0.19/0.43 |
| 6-Class | 35227.618 | 35755.980 | 35473.197 | 0.655 | 0.065 | <0.001 | 0.43/0.04/0.09/0.02/0.24/0.18 |
| 7-Class | 35204.079 | 35821.491 | 35491.048 | 0.582 | 0.218 | <0.001 | 0.17/0.04/0.24/0.02/0.18/0.10/0.25 |

AIC: Akaike Information Criterion.

BIC: Bayesian Information Criterion.

aBIC: Adjusted Bayesian Information Criterion.

LMR: Lo-Mendell-Rubin Likelihood Ratio Test.

BLRT: Bootstrap Likelihood Ratio Test.

Supplementary Table 3-1 LCA-based average posterior probabilities for members in each observed group (rows) belonging to each latent class (columns) based on 2020 data

| LCA Classification | Latent Classes | | | |
| --- | --- | --- | --- | --- |
|  | 1 | 2 | 3 | 4 |
| 1 | **0.782** | 0.020 | 0.160 | 0.038 |
| 2 | 0.065 | **0.762** | 0.112 | 0.061 |
| 3 | 0.168 | 0.060 | **0.766** | 0.005 |
| 4 | 0.040 | 0.062 | 0.016 | **0.882** |

The higher the values on the diagonal, the higher the classification accuracy.

Supplementary Table 3-2 LCA-based classification probabilities for members of each latent class (rows) being assigned to each observed group (columns) based on 2020 data

| Latent Classes | LCA Classification | | | |
| --- | --- | --- | --- | --- |
|  | 1 | 2 | 3 | 4 |
| 1 | **0.824** | 0.016 | 0.152 | 0.007 |
| 2 | 0.078 | **0.682** | 0.198 | 0.042 |
| 3 | 0.189 | 0.031 | **0.777** | 0.003 |
| 4 | 0.179 | 0.067 | 0.021 | **0.734** |

Classification accuracy increases with higher values along the diagonal.

Supplementary Table 4 Confusion matrix and performance metrics of KNN classification model based on 2020 data

1. Confusion Matrix

| Predicted \ Actual | Class 1 | Class 2 | Class 3 | Class 4 |
| --- | --- | --- | --- | --- |
| Class 1 | **359** | 5 | 9 | 2 |
| Class 2 | 2 | **79** | 2 | 0 |
| Class 3 | 8 | 2 | **306** | 0 |
| Class 4 | 0 | 0 | 0 | **63** |

1. Performance Metrics

| Metric | Class 1 | Class 2 | Class 3 | Class 4 |
| --- | --- | --- | --- | --- |
| Sensitivity | 0.973 | 0.919 | 0.965 | 0.969 |
| Specificity | 0.966 | 0.995 | 0.981 | 1.000 |
| Positive Predictive Value | 0.957 | 0.952 | 0.968 | 1.000 |
| Negative Predictive Value | 0.978 | 0.991 | 0.979 | 0.997 |
| Accuracy | 0.964 | | | |
| Classification Error Rate | 0.036 | | | |
| Kappa | 0.944 | | | |

Supplementary Table 5-1 Initial Probability Matrix of Four Multimorbidity Patterns (five waves, n (%))

| Time Point | Multi-system Disorder Group | Gastrointestinal Metabolism Group | Cardiovascular Disease Group | Respiratory System Disease Group |
| --- | --- | --- | --- | --- |
| 2011 | 155(5.54%) | 2090(74.70%) | 444(15.87%) | 109(3.90%) |
| 2013 | 201(7.18%) | 1948(69.62%) | 529(18.91%) | 120(4.29%) |
| 2015 | 198(7.08%) | 1722(61.54%) | 606(21.66%) | 272(9.72%) |
| 2018 | 293(10.47%) | 1408(50.32%) | 869(31.06%) | 228(8.15%) |
| 2020 | 289(10.33%) | 1233(44.07%) | 1058(37.81%) | 218(7.79%) |

Supplementary Table 5-2 Transition Frequency Matrix for 2011-2013 (%)

| From \ To | Multi-system Disorder Group | Gastrointestinal Metabolism Group | Cardiovascular Disease Group | Respiratory System Disease Group |
| --- | --- | --- | --- | --- |
| Multi-system Disorder Group | **92.90%** | 1.94% | 2.58% | 2.58% |
| Gastrointestinal Metabolism Group | 1.63% | **92.78%** | 5.22% | 0.38% |
| Cardiovascular Disease Group | 4.95% | 1.35% | **93.69%** | 0.00% |
| Respiratory System Disease Group | 0.92% | 0.00% | 0.00% | **99.08%** |

Bold values indicate the proportion of individuals who remained in the same multimorbidity pattern during the transition.

Supplementary Table 5-3 Transition Frequency Matrix for 2013-2015 (%)

| From \ To | Multi-system Disorder Group | Gastrointestinal Metabolism Group | Cardiovascular Disease Group | Respiratory System Disease Group |
| --- | --- | --- | --- | --- |
| Multi-system Disorder Group | **50.75%** | 22.89% | 20.90% | 5.47% |
| Gastrointestinal Metabolism Group | 2.36% | **82.49%** | 7.29% | 7.85% |
| Cardiovascular Disease Group | 5.29% | 13.04% | **79.77%** | 1.89% |
| Respiratory System Disease Group | 18.33% | 0.00% | 0.00% | **81.67%** |

Bold values indicate the proportion of individuals who remained in the same multimorbidity pattern during the transition.

Supplementary Table 5-4 Transition Frequency Matrix for 2015-2018 (%)

| From \ To | Multi-system Disorder Group | Gastrointestinal Metabolism Group | Cardiovascular Disease Group | Respiratory System Disease Group |
| --- | --- | --- | --- | --- |
| Multi-system Disorder Group | **55.05%** | 20.71% | 10.10% | 14.14% |
| Gastrointestinal Metabolism Group | 3.72% | **69.40%** | 23.81% | 3.08% |
| Cardiovascular Disease Group | 16.17% | 13.37% | **68.48%** | 1.98% |
| Respiratory System Disease Group | 8.09% | 33.46% | 8.82% | **49.63%** |

Bold values indicate the proportion of individuals who remained in the same multimorbidity pattern during the transition.

Supplementary Table 5-5 Transition Frequency Matrix for 2018-2020 (%)

| From \ To | Multi-system Disorder Group | Gastrointestinal Metabolism Group | Cardiovascular Disease Group | Respiratory System Disease Group |
| --- | --- | --- | --- | --- |
| Multi-system Disorder Group | **56.66%** | 10.24% | 27.65% | 5.46% |
| Gastrointestinal Metabolism Group | 3.34% | **75.28%** | 18.54% | 2.84% |
| Cardiovascular Disease Group | 5.98% | 12.08% | **80.67%** | 1.27% |
| Respiratory System Disease Group | 10.53% | 16.67% | 6.58% | **66.23%** |

Bold values indicate the proportion of individuals who remained in the same multimorbidity pattern during the transition.
